# Application of information theory in systems biology

**DOI:** 10.1007/s12551-020-00665-w

**Published:** 2020-03-06

**Authors:** Shinsuke Uda

**Affiliations:** Division of Integrated Omics, Research Center for Transomics Medicine, Medical Institute of Bioregulation, Kyushu University, 3-1-1 Maidashi, Higashi-ku, Fukuoka, 812-8582 Japan

**Keywords:** Information processing, Systems biology, Information theory

## Abstract

Over recent years, new light has been shed on aspects of information processing in cells. The quantification of information, as described by Shannon’s information theory, is a basic and powerful tool that can be applied to various fields, such as communication, statistics, and computer science, as well as to information processing within cells. It has also been used to infer the network structure of molecular species. However, the difficulty of obtaining sufficient sample sizes and the computational burden associated with the high-dimensional data often encountered in biology can result in bottlenecks in the application of information theory to systems biology. This article provides an overview of the application of information theory to systems biology, discussing the associated bottlenecks and reviewing recent work.

## Introduction

Systems biology has contributed greatly to our understanding of life phenomena by helping elucidate their underlying mechanisms from a systems perspective. Various systems approaches can be applied, such as the consideration of mechanics or of biochemical reactions, but in recent years there has been increased focus on information processing in cells (Uda and Kuroda 2016) (Levchenko and Nemenman 2014) (Tkačik and Bialek 2016). That is not to say that investigating cells as information processing systems is entirely novel; in neuroscience, there have been various studies of the mechanisms of information processing in neurons and neuronal networks (Rieke et al. 1997; Timme and Lapish 2018), and the notion of information processing in cells, especially cellular signal transduction, has previously been described (Azeloglu and Iyengar 2015).

In most cells, information processing is implemented by biochemical processes; in neurons, it is implemented through electrical signaling. Collecting quantitative data is generally more difficult for biochemical processes than for the electrical signals in neurons, and this has hampered the analysis of information processing in cells. However, recent developments in technology have made it possible to quantitatively measure various biochemical processes, and thus to investigate information processing, in a single cell (Cheong et al. 2011; Gregor et al. 2007; Keshelava et al. 2018; Ozaki et al. 2010; Selimkhanov et al. 2014).

Quantitative analysis of information processing requires a definition of the amount of information. This is provided by Shannon’s information theory (Cover and Thomas 2006; Shannon 1948). In this theory, information is defined and formulated in the context of communication between a sender and receiver; the definitions are general and can be applied not only to communication, but also to fields such as statistics, machine learning, computer science, and gambling. Systems biology is no exception; the application of Shannon’s information theory and its definition of information allows the quantitative analysis of mechanisms of information processing. However, this raises some concerns in terms of sample size, the dimension of the data vector, and the interpretation of the analysis results. In this article, the author describes the efficacy of applying Shannon’s information theory to systems biology and discusses notable points specific to biological situations.

## Information quantification

Data have become increasingly important in this Information Age. Data sets are generally used with the expectation they contain useful information, but how can the amount of information they contain be quantified?

Consider data sampled from a statistical population, where a random variable *X* is generated from a distribution and the outcome *x* of *X* is a part of the data. All information on *X* is contained in the distribution of *X* because *x* can be generated if the distribution is known. Intuitively, the amount of information contained might be expected to be proportional to the length of the sequence representing the data items sampled from the population. However, this is not necessarily the case. For example, take the example of a coin flip, where *x* = 1 and *x* = 0 represent an outcome of heads and tails, respectively. The data set of the observed outcomes for coin flips repeated *n* times can be written as a sequence of length *n*: ${\left\{{x}_i\right\}}_{i=1}^n$. If, however, the representations of heads and tails are changed to *x* = 11 and *x* = 00, this doubles the length of the sequence. Similarly, the length of the sequence can be increased without limit through the redundant representation of the data, with no change in the amount of information contained in the data. It therefore seems to be essential that measuring the amount of information contained in a data set requires non-redundant representation of the data.

Let *l*_*i*_ be the description length of event *i*, which occurs with probability *p*_*i*_. The average description length of the data has the following lower bound (Cover and Thomas 2006):

1 $$ \sum \limits_i{p}_i{l}_i\ge -\sum \limits_i{p}_i\log {p}_i\equiv H(p) $$

This lower bound corresponds to the entropy *H* of distribution {*p*_*i*_}. Inequality (1) means that the average description length for arbitrary representation of event *i* cannot be reduced to less than the value of entropy. In the case of a continuous random variable, the summation is replaced with the equivalent integral. Thus, entropy can be used as a measure to quantify the amount of information contained in a distribution. The most commonly used unit of information is the “bits,” which represents the length of a binary sequence, because most data can be transformed to binary representation.

Equation (1) indicates that a data set of length *n* with entropy *H*(*p*) has an average number of states of 2^*nH*(*p*)^, where the base of logarithms is 2 and the unit of entropy is bits. Thus, the entropy can also be interpreted as an index of uncertainty, with larger values indicating greater uncertainty. It may not be immediately intuitive that a measure of uncertainty would be an indicator of the amount of information; however, it may help to keep in mind that uncertainty is defined by the average number of states.

**Fig. 1:**
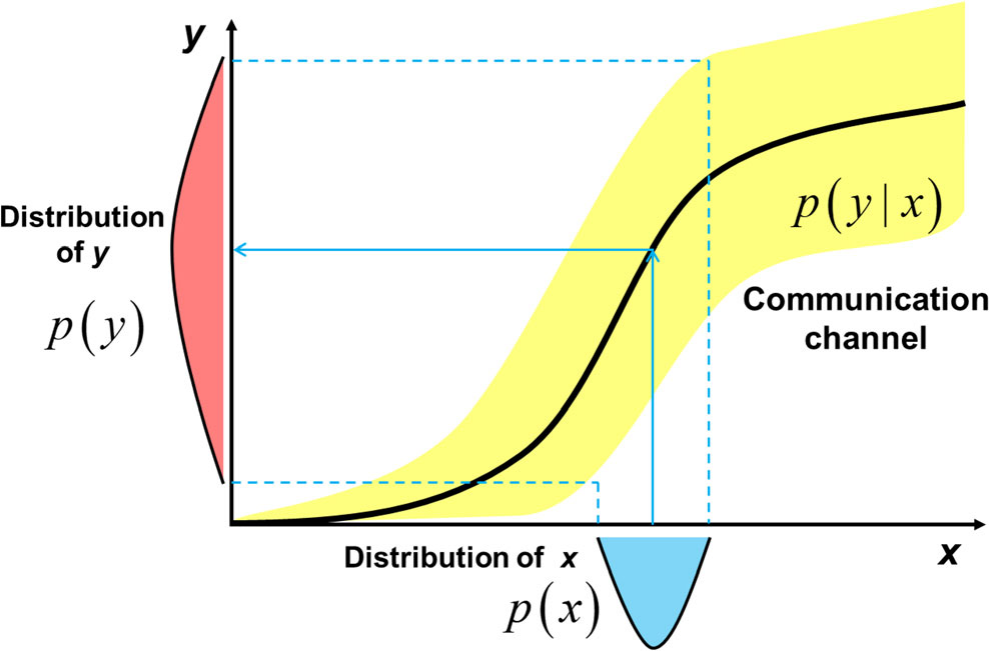
The relationship of the distributions of *x* as the sender and *y* as the receiver. The solid line is the average of *y* given *x*. The vertical height of the yellow area for a given value of *x* is the variability of *y* given *x*

**Fig. 2:**
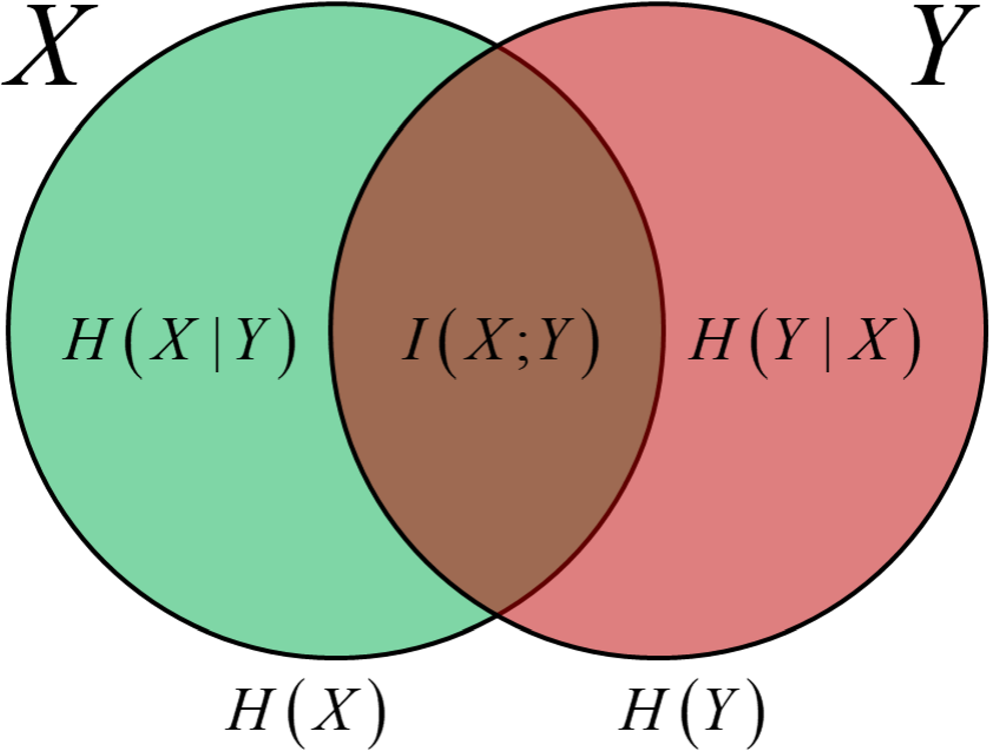
Graphical representation of entropy and mutual information. The circles represent the entropies of the random variables *X* and *Y*. The area of intersection of the two circles corresponds to the mutual information between the variables. The remainder of each circle outside the intersection corresponds to each conditional entropy

Consider a sender transmitting a message to a receiver. How can the amount of information reliably transmitted between the sender and receiver be quantified? Information transmission is measured according to the mutual information to the sender’s and receiver’s data *X* and *Y*, respectively, as follows:

2 $$ {\displaystyle \begin{array}{l}I\left(X;Y\right)=H(X)-H\left(X|Y\right)=H(Y)-H\left(Y|X\right)\\ {}=\sum \limits_{x,y}p\left(x,y\right)\log \frac{p\left(y|x\right)}{p(y)}\end{array}} $$

where *p*(⋅) indicates a probabilistic distribution function. The conditional distribution *p*(*y*| *x*) is termed a communication channel in the context of communication, and defines the distribution of *Y* given *X*; this can be interpreted as the relationship between *X* as the input and *Y* as the output (Fig. 1). The term 2^*I*(*X*;*Y*)^, based on the mutual information *I*(*X*;*Y*), corresponds to the average number of states of the sender’s data that the receiver can distinguish. The mutual information can be described by the difference between the entropy and the conditional entropy (Fig. 2), where the conditional entropy *H*(*A*| *B*) is the amount of information of *A* when *B* is known. Thus, the mutual information *I*(*X*; *Y*) can be interpreted as the information that is the residue of information *X* (or *Y*) omitted the information when *Y* (or *X*) is known. Schematically, this corresponds to the intersection of the information content of *X* and *Y*. If *I*(*X*; *Y*) = 0, there is no information transmission between the sender and the receiver. This is equivalent to *X* and *Y* being statistically independent.

Given the distributions *p*(*x*) and *p*(*y*| *x*), the mutual information is uniquely determined by Eq. (2) (Fig. 1), where *p*(*x*, *y*) = *p*(*x*)*p*(*y*| *x*) and $p(y)=\sum \limits_xp\left(y|x\right)p(x)$. In the transmission process, the channel *p*(*y*| *x*) typically depends on the communication system used, but the input distribution *p*(*x*) can often be designed. The upper bound for the mutual information, given an input distribution *p*(*x*), is known as the channel capacity:

$$ C=\underset{p(x)}{\max }I\left(X;Y\right) $$

When applying these concepts to biological situations, the in vivo input distribution is often difficult to measure, so the channel capacity is often calculated instead of evaluating the mutual information from the input distribution.

Although, for simplicity, *X* and *Y* have so far been denoted as scalar quantities, they can more generally be represented as vectors. When the data form a time series, the information including time effects can be represented by the extension of random variables such as *X* and/or *Y* from scalar to vector. For simplicity, consider time to be discrete rather than continuous, given by *t* = {*t*_*i*_}; then, the time trajectory of *X* can be represented as {*X*_*t*_} = (*X*_1_, ⋯, *X*_*m*_)^*T*^. The mutual information between time trajectory {*X*_*t*_} and {*Y*_*t*_} is given by:

3 $$ I\left(\left\{{X}_t\right\};\left\{{Y}_t\right\}\right)=\sum \limits_{\left\{{x}_t\right\},\left\{{y}_t\right\}}p\left(\left\{{x}_t\right\},\left\{{y}_t\right\}\right)\log \frac{p\left(\left\{{y}_t\right\}|\left\{{x}_t\right\}\right)}{p\left(\left\{{y}_t\right\}\right)} $$

This can be considered to be the information transmission between a sender and receiver (Munakata and Kamiyabu 2006). The mutual information of the snapshot at a single time given by Eq. (2) corresponds to the lower bound of the mutual information of the time trajectory given by Eq. (3).

The mutual information *I*(*X*; *Y*) does not always indicate the direct statistical interaction between *X* and *Y*. For example, if *Z* interacts with *X* and *Y*, and *X* and *Y* are not independent, then even if *X* and *Y* do not directly interact, there is an indirect interaction between *X* and *Y* mediated through *Z*. The conditional mutual information:

$$ I\left(X;Y|Z\right)=\sum \limits_{x,y,z}p\left(x,y,z\right)\log \frac{p\left(y|x,z\right)}{p\left(y|z\right)} $$

allows the statistical relationship between *X* and *Y*, subtracting the effect of *Z* to be quantified.

Transfer entropy (Hlavackova-Schindler et al. 2007; Palus et al. 2001; Schreiber 2000):

$$ {\displaystyle \begin{array}{l}\mathrm{TE}\left(X\to Y\right)=I\left({\left\{{y}_i\right\}}_{i=t+1}^T;{\left\{{x}_i\right\}}_{i=t}^{t-{\tau}_x}|{\left\{{y}_i\right\}}_{i=t}^{t-{\tau}_y}\right)\\ {}=\sum \limits_{{\left\{{y}_i\right\}}_{i=t+1}^T,{\left\{{y}_i\right\}}_{i=t}^{t-{\tau}_y},{\left\{{x}_i\right\}}_{i=t}^{t-{\tau}_x}}p\left({\left\{{y}_i\right\}}_{i=t+1}^T,{\left\{{y}_i\right\}}_{i=t}^{t-{\tau}_y},{\left\{{x}_i\right\}}_{i=t}^{t-{\tau}_x}\right)\log \frac{p\left({\left\{{y}_i\right\}}_{i=t+1}^T|{\left\{{y}_i\right\}}_{i=t}^{t-{\tau}_y},{\left\{{x}_i\right\}}_{i=t}^{t-{\tau}_x}\right)}{p\left({\left\{{y}_i\right\}}_{i=t+1}^T|{\left\{{y}_i\right\}}_{i=t}^{t-{\tau}_y}\right)}\end{array}} $$

is a specific application of conditional mutual information to the analysis of time series, where *T*, *τ*_*x*_ and *τ*_*y*_ indicate the number of lags. It can be interpreted as an extension of Granger causality to a non-linear relationship.

### Bottlenecks

In general, it is not easy to evaluate mutual information from data with a finite sample size. Especially in biology, sample sizes tend to be relatively small but with high dimensions. This results in bottlenecks and makes the evaluation of mutual information difficult.

Mutual information is defined by using a distribution function. Thus, the evaluation of mutual information requires the distribution function to be estimated directly or indirectly. However, estimating a distribution function from data is not easy in practice, especially in high dimensions (Hastie et al. 2009). A simple method for estimating a distribution function is by using a normalized histogram; however, the value of the resulting mutual information will vary depending on the bin size of the histogram, and selecting bin size is an unexpectedly troublesome task. The variance in the estimated distribution function increases as the bin size decreases, and its bias increases as the bin size increases. A number of methods to select bin size have been proposed (Freedman and Diaconis 1981; Scott 1979; Shimazaki and Shinomoto 2007; Sturges 1926). However, it is still not easy to control the bias–variance trade-off when selecting the bin size for practical data (Hastie et al. 2009). For high-dimensional data, the exponential increase in the number of bins makes it especially difficult to process the histogram.

An improved method for constructing histograms using B-spline functions has been proposed by Daub et al. (Daub et al. 2004). This method is more efficient than using an ordinary histogram for estimating a distribution function by the extension of bins to polynomial functions with the use of characteristics of B-spline functions, requiring the selection of the bin size and the order of the B-spline function. The kernel density estimation method (Parzen 1962) is frequently used to estimate a distribution function; this requires the selection of the bandwidth of the kernel function rather than the bin size. Various methods to select the bandwidth have been proposed (Turlach 1993), but there remains the problem of the bias–variance trade-off for the selection of bandwidth.

Estimating a distribution function with high accuracy and precision generally requires a sample size difficult to achieve in biology experiments. The sample size to be required to estimate distribution function increases exponentially as the number of dimensions increases, so it is especially difficult to reliably estimate a distribution function in high dimensions. The computational burden for evaluating information also increases exponentially because the summations in Eqs. (1)–(3) run over all the domains of the variables. This makes the straightforward computation of information in high dimensions an intractable problem. In biology, the number of molecular species is of the order 10^3^ to 10^5^, depending on the omics layer, and so the number of dimensions of biological data, especially omics data, is often large. However, reformulating the information equations in terms of the expectation of the logarithm of a distribution function or the ratio between distribution functions can be useful. When the distribution functions are known, the computation of the expectation for all domains can be approximated by the sample mean. This approximation by sample mean only needs the values of probability density at the sampling points; thus, estimating distribution function is not needed. This reduces the computational burden for the expectation to the order of the sample size, and in biology the sample size is relatively small compared with the number of dimensions. For example, in Eq. (2), the following holds:

$$ E\left[\log \frac{p\left(y|x\right)}{p(y)}\right]\approx \frac{1}{n}\sum \limits_i^n\log \frac{p\left({y}_i|{x}_i\right)}{p\left({y}_i\right)} $$

The Kozachenko–Leonenko estimator (Kozachenko and Leonenko 1987; Kraskov et al. 2004) can be used to compute the quantity of information based on approximations by using the sample mean and applying the *k*-nearest neighbors method, even for high-dimensional data. However, the accuracy of the Kozachenko–Leonenko estimator seems to be low, especially for high dimensions, and the information such as entropy and mutual information often take negative values. The parameter *k* for the number of nearest neighbors needs to be selected, but as yet there is no theoretical criterion on how to do this. In addition, there is the problem of the bias–variance trade-off for the selection of parameter *k*.

Thus, sample sizes and computational burden could be bottlenecks to evaluate the quantity of information.

## The application of information theory to systems biology

Information theory has contributed to systems biology in two main ways: the analysis of information transmission in cells and the inference of the network structure of molecular species. In the former, information theory has been used to quantify information transmission (previous studies are summarized in Table 1). In the latter, information theory has been used to examine the presence or absence of statistical relationships.

**Table 1:** Summary of previous studies on information transmission in biological systems (this table is modified from Uda and Kuroda (2016))

Authors	Measurement technique	Sender	Receiver	Biological System	Main result
Tkačik et al.	Snapshot	Bicoid	Hunchback	Transcription factor, gene expression	Comparing information transmission in vivo to channel capacity
Cheong et al.	Snapshot	TNF	NFĸB, ATF-2	Nuclear translocation, protein phosphorylation	Information transmission by multiple molecular species
Uda et al.	Snapshot	Growth factors, ERK, CREB	ERK, CREB, c-FOS, EGR1	Protein phosphorylation, gene production	Robustness and compensation of information transmission
Selimkhanov et al.	Live imaging	EGF	ERK	Protein phosphorylation, small molecule, nuclear translocation	Information transmission by temporal pattern
		ATP	Ca2+		
		LPS	NFĸB		
Keshelava et al.	Live imaging	Acetylcholine	Ca^2+^	G protein-coupled receptor signaling	Information transmission at a single cell level

In recent studies of information transmission, information theory has been used to examine signal transduction, considered to be a core mechanism in cellular information processing (Tkačik et al. 2008a, b; Cheong et al. 2011; Lestas et al. 2010; Levchenko and Nemenman 2014; Selimkhanov et al. 2014; Tostevin and ten Wolde 2009; Uda et al. 2013; Waltermann and Klipp 2011; Yu et al. 2008). Tkačik et al. examined the channel capacity and mutual information between an upstream transcription factor, Bicoid, and a downstream target gene product, Hunchback, during early embryogenesis in *Drosophila* flies (Tkačik et al. 2008b). As discussed earlier, mutual information is generally calculated from the input distribution and the conditional distribution. Unlike most biological experiments, in which the measurement of the input distribution in vivo is typically difficult, it is possible to measure the in vivo distribution of Bicoid concentrations (Gregor et al. 2007). Tkačik et al. reported that the mutual information was almost 1.5 bits, close to the channel capacity of almost 1.7 bits. These findings indicated that in vivo Bicoid/Hunchback system uses a distribution of the input, Bicoid, that results in the channel capacity. This is interesting, with the close agreement in the values of mutual information and the channel capacity possibly implying a design principle in cellular information processing.

Cheong et al. examined the channel capacity between upstream tumor necrosis factor and the downstream nuclear factor(NF) κB or activating transcription factor–2 (ATF-2) and found that information transmission increased through a combination of the effects of NFκB and ATF-2 (Cheong et al. 2011). In addition, they defined two models, the bush model and the tree model, which differ in terms of the network structure for information transmission. In the bush model, information is transmitted downstream by branched pathways directly. In the tree model, information is transmitted via the pathways, which go through a common downstream molecule. They investigated the characteristics of information transmission by comparing two models under the assumption of a Gaussian distribution.

Uda et al. examined channel capacities between growth factors and either signaling molecules or immediate early genes (Uda et al. 2013). They demonstrated that each channel capacity is almost 1 bit for each growth factor, but each growth factor uses a specific pathway to transmit information through the use of multivariate mutual information. In addition, the information transmission was generally more robust than the average signal intensity, despite pharmacological perturbations, and compensation for information transmission occurred. The mechanism or mathematical conditions underlying this robustness and compensation have not been fully elucidated; further study is needed. Robust information transmission has also been identified in the spines of neurons by numerical stochastic simulation; despite the signal being noisy, the information transmission in spines with small volumes is efficient compared with that for those with large volumes, and is sensitive to the input (Fujii et al. 2017; Tottori et al. 2019a, 2019b).

Tkačik et al., Cheong et al., and Uda et al. acquired data sets for estimating the distributions in their studies by snapshots taken at a series of time points. With this method, the combined effects of multiple time points are omitted from the calculation of mutual information, which results in the information transmission being underestimated. Generally, calculating mutual information and the channel capacity between a stimulus and the associated time trajectory, which consists of multiple time points, increases the computational difficulty because of the high-dimensional summation involved. Tostevin and ten Wolde theoretically analyzed the mutual information between time trajectories generated by biochemical reactions based on a Gaussian approximation (Tostevin and ten Wolde 2009).

Selimkhanov et al. measured the continuous time course of molecular concentrations by live imaging and efficiently calculated the mutual information and channel capacity between the stimulus and the associated time trajectory of downstream molecules, including the combined effect at multiple time points, by applying the *k*-nearest neighbors method (Selimkhanov et al. 2014). The combined effect can increase the channel capacity compared with using only a snapshot at a single time point, as can combining molecular species.

A method using machine learning for evaluating mutual information has been proposed (Cepeda-Humerez et al. 2019). In this, the time trajectories and inputs, which represent the stimuli and experimental conditions, are regarded as the explanatory and response variables, respectively. After training the model for classification or regression with a training data set, mutual information was estimated from the prediction error by using a test data set. This method is based on the intuitive idea that prediction error is reduced by increasing the amount of information the time trajectories include about the input corresponding to the stimulation.

**Fig. 3:**
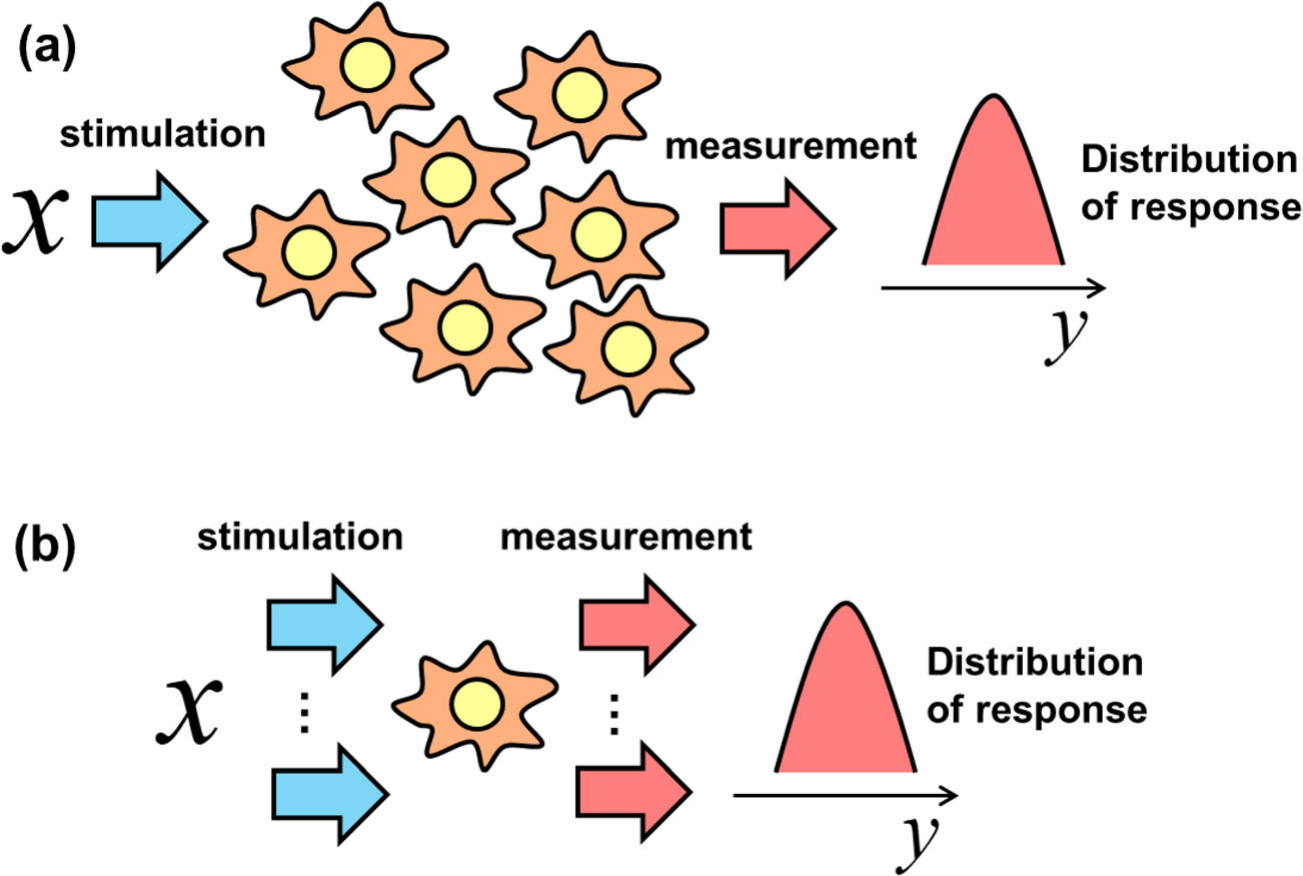
Schematic interpretation of information at the population level and information at the single cell level. **a** Information at the population level is evaluated from a distribution obtained from the responses of a population of cells where each cell is stimulated once. **b** Information at the single cell level is evaluated from a distribution obtained from the responses of a single cell stimulated repeatedly

The data to estimate information transmission is usually acquired by stimulating a single cell once. Because this estimate requires a large sample size for the data set, as discussed earlier, many single cells need to be measured. When this approach is used, as in the studies described above, the author refers here to the resulting information as “information at the population level” (Fig. 3a). Conversely, a single cell can be stimulated and measured repeatedly to obtain the data. This requires the characteristics of the single cell system to remain unchanged with the repeated stimulation and measurements. The author refers to the information collected in this way as “information at the single cell level” (Fig. 3b). Information at the population level assumes that the cell systems do not differ between the cells or that the response at the population level does not vary; this can be interpreted as the receiver not discriminating between the signals in specific single cells. Conversely, information at the single cell level assumes that the cell system can vary between cells. The interpretation of the information depends on the problem settings involved in encode and decode systems.

Keshelava et al. (Keshelava et al. 2018) repeatedly stimulated and measured the responses of single cells by using a live imaging technique, and evaluated the channel capacity at the single cell level. This varied between single cells, suggesting that the system of information transmission differs between individual cells. The average channel capacity at the single cell level (almost 2 bits) was larger than the channel capacity at the population level.

Information theory has also been employed to infer the network structure of molecular species. In this case, information is used to examine statistical relationships between molecular species. Several methods to infer the structures of biological networks have been reported. Here, the author focuses on those based on an information theory approach. The ARACNE (Margolin et al. 2006) and CLR (Faith et al. 2007) network inference algorithms determine the presence of an edge between nodes by calculating the mutual information of the two nodes. ARACNE employs data processing inequalities to eliminate the weakest associations in every closed triplet of nodes. Intuitively, suppose a relay of information transmission, data processing inequality means that the quantity of information of end point cannot increase more than that of relay point. This procedure is exact when the network has a tree structure. In contrast, CLR compares the values of mutual information for a particular pair of nodes to the background distribution, which is empirically estimated from the two sets of values of mutual information: the set of values of mutual information between one of the pairs and all nodes, and the set of values of mutual information between the other of the pairs and all nodes. CLR is based on the assumption that the empirical distribution provides background information about the absence of edges. On the other hand, a statistical hypothesis test also provides a threshold of mutual information to determine the absence of edges. Even when *X* and *Y* are independent, *I*(*X*; *Y*) = 0 does not always hold because of the sampling error resulting from the finite sample size. The permutation test is effective for examining the statistical significance of the null hypothesis *I*(*X*; *Y*) = 0 and the alternative hypothesis *I*(*X*; *Y*) ≠ 0 (Daub et al. 2004).

The non-negative decomposition of multivariate mutual information has been applied to the inference of network structure from single-cell transcriptome data (Chan et al. 2017). Transfer entropy has been used to infer the connectivity of a neuronal network from time series data of neuronal activity (Vicente et al. 2011; Terada et al. 2019). Entropy has also been used to characterize a population of differentiated cells (Grun et al. 2016).

## Summary and perspectives

Information theory is a powerful tool for quantifying information transmission in cells and inferring the network structure of molecular species and the connectivity of neuronal networks. The cost of data acquisition in biology can be high; nevertheless, the evaluation of quantity of information requires a large sample size because this is defined by distribution functions. In addition, when the data set is high dimensional, such as with omics data and time series, the computational burden increases exponentially with the increase in dimensions. When the sample size is small and the data are high dimensional, this can result in a bottleneck to applying information quantification; however, many computational methods are being developed to avoid such a bottleneck. It is important to choose suitable methods to evaluate the quantity of information on problem setting such as biological situations and experimental conditions. Bottlenecks could potentially be avoided by the future development of suitable experimental measurement techniques and computational methods, allowing information theory to be applied more widely to systems biology.

The interpretation of information transmission quantified by information quantification methods is not yet fully established, possibly because of its short research history. Information transmission refers essentially to the potential amount of information that can be transmitted. For example, if the information transmission of a pathway within a cell is 2 bits, this means that four states can be controlled by the pathway; however, the cell needs to control only two states of survival or differentiation. The value of mutual information does not always correspond to the amount of biologically meaningful information. The elucidation of information transmission linked with a decoding system is needed to clarify the biological meaning of information.

The development of computational methods for information transmission on a time trajectory would allow the examination of how to encode information to a time trajectory. For example, epidermal growth factor stimulation has been reported to induce the transient phosphorylation of extracellular signal-regulated kinase (pERK) and cell proliferation, whereas nerve growth factor stimulation induces the sustained production of pERK and cell differentiation (Marshall 1995; Gotoh et al. 1990; Qiu and Green 1992; Traverse et al. 1992). This means that information for the distinct growth factors is encoded into the specific time trajectory of pERK, which is selectively decoded by the downstream pathways, resulting in the appropriate cell fate decisions (Sasagawa et al. 2005). This suggests that the time trajectories for the transient and sustained conditions enhance the information transmission. Extracting information about the enhancing part or pattern of such time trajectories could help elucidate the encoding mechanism underlying information transmission.

A drawback of information theory in the inference of network structures of molecular species is the difficulty of inferring the direction of edges because information quantification is symmetric for *X* and *Y*. One method for inferring the direction of edges is to use the transfer entropy. Although it remains difficult to infer the structure of a large network, such as in an omics data set, the structure of a partial network can be inferred from a time series data set of multiple molecular species.

Currently, the main contribution of studies of information transmission is limited to cellular signal transduction. However, the elucidation of the entire spectrum of life phenomena across multi-omic layers, that is, by using transomics analysis, is attracting research attention (Yugi et al. 2016; Yugi and Kuroda 2018). However, the transomics analysis of information transmission is difficult under the present circumstances because of the difficulty of data acquisition, the high-dimensional nature of the data, and the multiple timescales involved. Nevertheless, from the long-term perspective, elucidation of how information is transmitted across multi-omic layers would be highly interesting. The author expects that the future development of measurement technology and analysis methods based on information theory could address this problem.
